# Obesity is associated with reduced cerebral blood flow – modified by physical activity

**DOI:** 10.1016/j.neurobiolaging.2021.04.008

**Published:** 2021-09

**Authors:** Silvin P. Knight, Eamon Laird, Wilby Williamson, John O’Connor, Louise Newman, Daniel Carey, Celine De Looze, Andrew J. Fagan, Michael A. Chappell, James F. Meaney, Rose Anne Kenny

**Affiliations:** a The Irish Longitudinal Study on Ageing (TILDA), Trinity College Dublin, Dublin, Ireland; b School of Medicine, Trinity College Dublin, Dublin, Ireland; c The Global Brain Health Institute (GBHI), Trinity College Dublin, Dublin, Ireland; d Department of Radiology, Mayo Clinic, Rochester, MN, USA; e Wellcome Centre for Integrative Neuroimaging, University of Oxford, Oxford, UK; f Institute of Biomedical Engineering, University of Oxford, Oxford, UK; g The National Centre for Advanced Medical Imaging (CAMI), St. James’s Hospital, Dublin, Ireland; h Mercer’s Institute for Successful Ageing (MISA), St. James’s Hospital, Dublin, Ireland

**Keywords:** Cerebral blood flow, Obesity, Physical activity, Cerebral perfusion, Arterial spin labeling MRI

## Abstract

This study examined the associations of body mass index (BMI), waist-to-hip ratio (WHR), waist circumference (WC), and physical activity (PA) with gray matter cerebral blood flow (CBF_GM_) in older adults. Cross-sectional data was used from the Irish Longitudinal Study on Ageing (n = 495, age 69.0 ±7.4 years, 52.1% female). Whole-brain CBF_GM_ was quantified using arterial spin labeling MRI. Results from multivariable regression analysis revealed that an increase in BMI of 0.43 kg/m^2^, WHR of 0.01, or WC of 1.3 cm were associated with the same reduction in CBF_GM_ as 1 year of advancing age. Participants overweight by BMI or with high WHR/WC reporting low/moderate PA had up to 3 ml/100g/min lower CBF_GM_ (*p* ≤ .011); there was no significant reduction for those reporting high PA. Since PA could potentially moderate obesity/CBF associations, this may be a cost-effective and relatively easy way to help mitigate the negative impact of obesity in an older population, such as cerebral hypoperfusion, which is an early mechanism in vascular dementia and Alzheimer’s disease.

## Highlights

• Increased BMI, WHR, and waist size associated with lower cerebral blood flow.
• Waist size +1cm associated with same reduction in cerebral blood flow as +1year age.
• Higher levels of physical activity shown to potentially modify these associations.

## Graphical abstract

**Figure:**
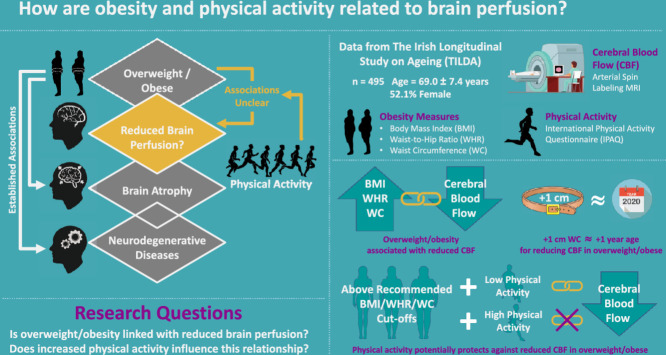

## 1 Introduction

The prevalence of obesity has doubled since 1980, with almost one-third of the global population now classified as overweight or obese. (Afshin et al., 2017; Koroukian et al., 2019) Obesity constitutes a significant public health issue given its adverse effect on physiological function. Compounding this, over the last decades there has also been a profound shift in the aging demographic, with a substantial increase in life expectancy. Obesity has been shown to increase the risk of several age-related conditions, including cardiovascular disease,(Czernichow et al., 2011; Larsson et al., 1984; Singh et al., 2013) brain atrophy,(Debette et al., 2010; Hamer and Batty, 2019; Kurth et al., 2013; Raji et al., 2010; Taki et al., 2008) and neurodegenerative conditions, such as dementia and Alzheimer’s disease.(Anstey et al., 2011; Gustafson et al., 2003; Kivipelto et al., 2005; Razay and Vreugdenhil, 2005) However, the potential neurovascular mechanisms underlying these associations are not well understood. It has also been suggested that the associations between obesity and health outcomes may be partly explained by specific health effects of different fat depot types. Several anthropometric measures have been proposed to quantify obesity; body mass index (BMI) is thought to be more reflective of fat stored peripherally, whereas waist-to-hip ratio (WHR) and waist circumference (WC) are indicative of fat located viscerally, with visceral fat potentially constituting a greater risk factor for heart disease and brain atrophy.(Emdin et al., 2017; Hamer and Batty, 2019)

While previous studies have suggested that obesity in early and midlife may be linked with reduced cerebral perfusion and blood flow velocity,(Willeumier et al., 2011; Williamson et al., 2018) little is known about these associations in later life.(Birdsill et al., 2013) Given the increased interest in altered brain structure and metabolism with aging, particularly in relation to dementia and Alzheimer’s disease,(Alexander et al., 2002; Buckner et al., 2005) examining how obesity may modify cerebral haemodynamic behaviour is an important area of study. Additionally, investigating these associations in an older cohort may help in understanding the mechanisms that underlie the onset of these neurodegenerative conditions as a person ages. Physical inactivity is also associated with many chronic conditions,(Lee et al., 2012) a reduction in executive function,(Daly et al., 2014; Peven et al., 2018) premature mortality,(Carlson et al., 2018) as well as increased risk of dementia and Alzheimer’s disease.(Aarsland et al., 2010; Laurin et al., 2001) In a clinical setting, beneficial effects of physical fitness interventions on cognitive performance have been reported in older persons.(Peig-Chiello et al., 1998; Satoh et al., 1995) Epidemiological studies have also reported that exercise may be protective for dementia and Alzheimer’s disease in older populations.(Laurin et al., 2001; Li et al., 1989) Whether any potential associations of obesity with cerebral blood flow could be modified by physical activity (PA) remains unclear.

In this study, the associations between anthropometric obesity indicators (BMI, WHR and WC) and gray matter cerebral blood flow (CBF_GM_), as measured using pseudo-continuous arterial spin labeling (pCASL) MRI, were examined in a large sample (n = 495) of older adults from The Irish Longitudinal Study on Ageing (TILDA). This study also investigated whether any potential associations between obesity and CBF_GM_ may be modified via higher levels of PA.

## 2 Methods

### 2.1 Design, setting and participants

This research was carried out as part of TILDA, an ongoing nationally-representative prospective cohort study of community-dwelling Irish adults (1 in 150 individuals in Ireland aged ≥50 years) established in 2009 (N = 8,507).(Donoghue et al., 2018; Kearney et al., 2011; Whelan and Savva, 2013) TILDA collects a wide range of health, economic and social data and investigates how these various factors interact; to date TILDA has completed five waves of data collection. The primary exposure variables for this study were measured at Wave 3 of TILDA (March 2014 − April 2016). Of 4,309 participants attending for health assessment in a dedicated health assessment centre, a random subset was invited to return for multi-parametric brain MRI. Participants were excluded if they reported a contraindication to MRI or a prior stroke/head injury. MRIs were performed between May 2014 and June 2015 at the National Centre for Advanced Medical Imaging, Dublin, Ireland. The mean (SD) delay between health assessment and MRI examination was 62 (40) days.(Donoghue et al., 2018) Final data collection was completed on 11th April 2016.

### 2.2 Standard protocol approvals, registration and patient consents

Ethical approval was granted for each wave of TILDA health assessment from the Health Sciences Research Ethics Committee at Trinity College Dublin, Dublin, Ireland, and all participants provided written informed consent. Additional ethics approval was received for the MRI sub-study from the St James’s Hospital/Adelaide and Meath Hospital, inc. National Children’s Hospital, Tallaght Research Ethic Committee, Dublin, Ireland. Those attending for MRI were also required to complete an additional MRI-specific consent form. All research was performed in accordance with the Declaration of Helsinki.

### 2.3 Brain imaging and analysis

In total, 578 participants attended for MRI though 18 did not provide data (due to claustrophobia/anxiety [n = 14] or MRI contraindication [n = 4]). MRI data were acquired using a 3T system (Achieva, Philips Medical Systems, The Netherlands) and a 32-channel head coil. The multi-parametric protocol included pCASL perfusion and *T*_1_-weighted structural acquisitions. Proton density (PD) images and magnetic field (*B*_0_) maps were also acquired.

The primary outcome for this study was CBF_GM_, quantified as the rate of delivery of arterial blood to brain tissue measured using pCASL-MRI. pCASL data were acquired using a total of 30 interleaved pairs of images acquired sequentially in a caudocranial direction, alternating with and without arterial spin labeling using two-dimensional multi-slice single shot echo-planar imaging (EPI) with background suppression (repetition time (TR)/echo time (TE) = 4,000/9 ms; flip angle (FA) = 90°; field-of-view (FOV) = 240 × 240 mm^2^; voxel size = 3 × 3 mm^2^; 13 slices (8mm thick, 1 mm gap); parallel imaging factor (SENSE) = 2.5; total scan duration = 4:16). The magnetic inversion plane was positioned in the neck, 90 mm caudal to the imaging volume. The labeling duration was 1,800 ms with a post-labeling delay (PLD) of 1,800 ms (the time separating the end of the labeling pulse and the start of image acquisition), as recommended in the ASL International Society for Magnetic Resonance in Medicine (ISMRM) white paper.(Alsop et al., 2015) A PLD of 1,800 ms was chosen in order to minimize any large vessel arterial signal in ASL images while maximizing the signal-to-noise ratio.(Haller et al., 2016) PD images were acquired with the same geometry as the pCASL acquisition (TR/TE = 10,000/9 ms; total scan duration = 20 s). *B*_0_ field maps were also measured using a two-echo two-dimensional gradient echo sequence with the same in-plane resolution as the pCASL scans (TR/TE_1_/TE_2_ = 455/1.69/7.0 ms; FA = 90°; 38 slices (3.2 mm slice thickness, 0.3 mm slice gap); total scan duration = 39 s). *T*_1_-weighted images were acquired using a three-dimensional magnetization-prepared rapid gradient echo (MP-RAGE) sequence (TR/TE = 6.7/3.1 ms; FA = 8°; FOV = 240 × 240 × 162mm^3^; voxel size = 0.8 × 0.8 × 0.9 mm^3^; SENSE = 2; total scan duration = 5:24). *T*_1_-weighted datasets were acquired from 560 participants, and pCASL data from 546 participants (attrition due to claustrophobia/inability to complete scan [n = 14]).

MRI analysis was performed using Oxford_ASL (v.4.0) (*FMRIB Software Library, FSL*; The University of Oxford, UK) in the FMRIB Software Library.(Smith et al., 2004) Firstly, brain extraction and tissue segmentation were performed using the *T*_1_-weighted images. Next, *B*_0_ maps were used to correct the EPI-acquired pCASL data for any spatially nonlinear distortion effects deriving from *B*_0_ inhomogeneities. Subtraction of the pCASL label-control pairs was then performed to generate perfusion-weighted images. Calibration was performed using cerebrospinal fluid (CSF) as a ‘reference-tissue’ (measured in the ventricles) and correction was made to the TR values used based on an assumed *T*_1_ value for CSF (4,300 ms), as well as the differences in *T*_2_ values between tissue (150 ms) and CSF (750 ms). Voxel-wise absolute perfusion values (cerebral blood flow (CBF) in ml/100g/min) were then calculated using the calibration (PD) data. A standard well-mixed single compartment kinetic model with no dispersion of the bolus of labeled blood water was used.(Buxton, 2005) A tissue *T*_1_ value of 1,300 ms and an arterial blood *T*_1_ of 1,650 ms were assumed.(Alsop et al., 2015) Slice-timing effects were also corrected for using a slice delay of 30 ms. Finally, a rigid registration from pCASL to structural space was performed and whole gray matter CBF was subsequently calculated. In order to create a gray matter mask, the gray matter partial volume effects from the structural segmentation were transformed into pCASL space and a threshold of 70% gray matter applied.(Chappell et al., 2017)

**Fig. 1:**
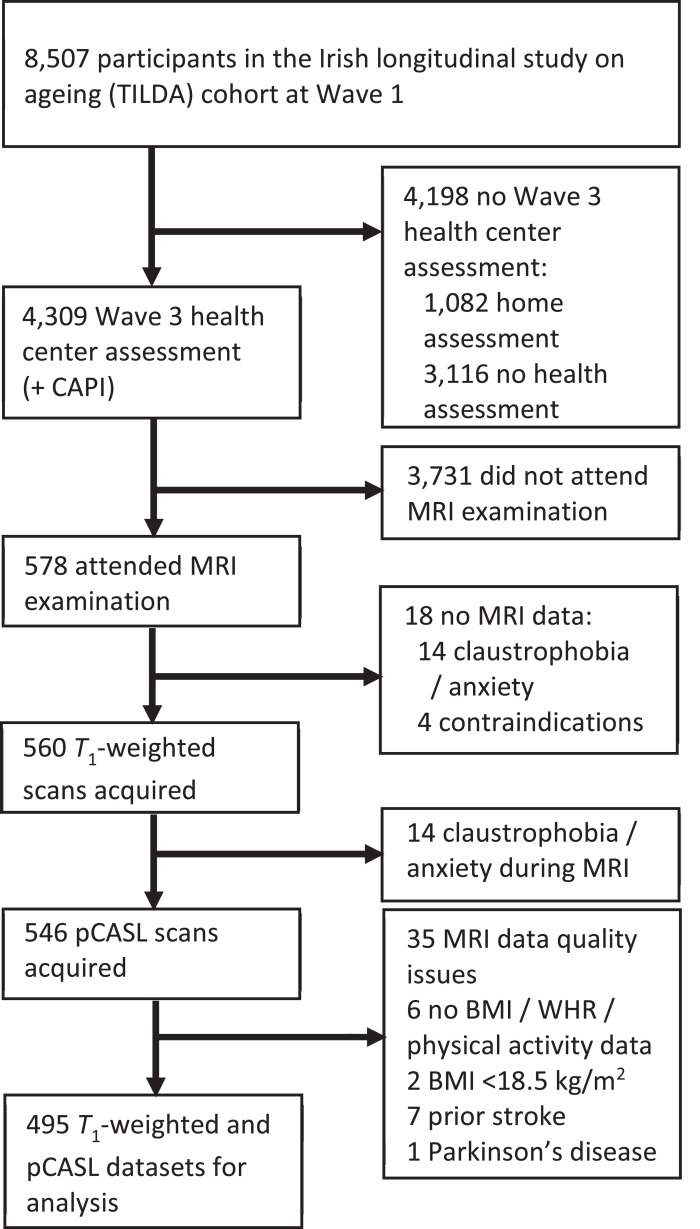
Flow chart describing sample selection and exclusions. Abbreviations: CAPI, computer assisted personal interview; pCASL, pseudo-continuous arterial spin labeling; BMI, body mass index; WHR, waist-to-hip ratio.

One participant was excluded from this study due to self-reported Parkinson’s disease and seven because of self-reported history of stroke. Two participants were excluded with BMI < 18.5 kg/m^2^ (‘underweight’). Six participants were excluded due to lack of either BMI, WHR or PA data. Two trained operators, who were blind to participant identity, reviewed all pCASL perfusion maps for evidence of arterial artefact, poor labelling of a feeding artery, severe motion and/or other gross failure to produce a perfusion image. By consensus between both operators, 27 subjects with abnormal perfusion maps were removed from the cohort. Of these, 16 were excluded due to labelling failure, four due to delayed arrival and seven due to severe motion. Another trained operator, who was blind to participant identity, screened all *T*_1_-weighted images for artefacts and/or pathology. A further eight subjects were excluded due to abnormalities on *T*_1_-weighted imaging. Among those, five had gross abnormality corresponding to confluent white matter hyperintensities on *T*_2_ and FLAIR imaging, two had established large vessel infarcts and one had MRI evidence of prior contusion/haemorrhage. In total 495 *T*_1_-weighted and pCASL datasets were included for analysis. Exclusion criteria leading to the sample are illustrated in Fig. 1.

### 2.4 TILDA measures

The comprehensive TILDA health assessment (previously described in detail [26]) included anthropometric measurements of height to the nearest 0.01m (Seca 240 Stadiometer, Seca Ltd, Birmingham, UK), weight to the nearest 0.1 kg (Seca 861 Electronic Scales, Seca Ltd, Birmingham, UK). Waist and hip circumferences were recorded to the nearest 0.01 m using a flexible tape measure (Seca Ltd, Birmingham, UK). Hip measurements were made at the level of the symphysis pubis and the waist measurements at the midpoint between the lower margin of the last palpable rib and the top of the iliac crest. Prior to each measure being taken, TILDA participants were requested to remove any heavy garments, footwear or head wear for measurement accuracy. BMI was calculated from the formula weight [kg] / (height [m])^2^. ‘Underweight’ was defined as BMI [kg/m^2^] <18.5, ‘normal’ as ≥18.5 and <25, ‘overweight’ as ≥25 and <30, and ‘obese’ as ≥30. (1995) WHR was calculated by dividing the separate waist and hip measurements from each participant. WHR cut-offs were set at ≥0.85 for females and ≥0.9 for males.(Consultation, 2008) WC cut-offs were set at ≥80 cm for females and ≥94 cm for males.(Consultation, 2008)

PA was quantified via the short form of the international physical activity questionnaire (IPAQ).(Craig et al., 2003) Participants were asked to report the number of days and typical time-per-day spent walking or doing moderate/vigorous intensity physical activities during the previous week. Participant were then subsequently classified as having ‘Low’, ‘Moderate’, or ‘High’ PA levels based on the criteria outlined in Table 1. Additionally, self-reported: educational attainment, cardiovascular conditions (angina; high blood pressure; heart: attack ever, failure, murmur, abnormal rhythm; high cholesterol; transient ischemic attack ever), diabetes, alcohol consumption habits (CAGE),(Ewing, 1984) smoking history, and anti-hypertensive medication use (coded using the Anatomical Therapeutic Chemical Classification (ATC): antihypertensive medications (ATC C02), diuretics (ATC C03), β-blockers (ATC C07), calcium channel blockers (ATC C08), and renin-angiotensin system agents (ATC C09)) were also recorded. Depressive symptoms were assessed using the Center for Epidemiologic Studies Depression scale (CESD).(Lewinsohn et al., 1997) Cognitive performance was also assessed via the Mini Mental State Examination (MMSE), though this was not included as a control variable in the models used in this study.

**Table 1:** Details of International Physical Activity Questionnaire (IPAQ) physical activity classifications (Craig et al., 2003)

Physical Activity Classifications	
High Activity	Any one of the following two criteria: • Vigorous* intensity activity on three or more days week accumulating at least 1,500 MET-minutes/week OR • Any combination of walking, moderate⁎⁎ or vigorous* intensity activities on seven days per week accumulating at least 3,000 MET-minutes/week
Moderate Activity	Any one of the following three criteria: • Vigorous* intensity activity of at least 20 min on three or more days per week OR • Moderate⁎⁎ intensity activity of at least 30 min on 5 or more days per week OR • Any combination of walking, moderate⁎⁎ or vigorous* intensity activities on five or more days per week accumulating at least 600 MET-minutes/week
Low Activity	Meeting none of the criteria for high or moderate activity

⁎ Vigorous activities require hard physical effort, resulting in breathing much harder than normal and can include heavy lifting, digging, aerobics or fast cycling.

⁎⁎ Moderate activities require moderate physical effort resulting in breathing somewhat harder than normal, for example carrying light loads, cycling at regular pace or doubles tennis.

### 2.5 Statistical analysis

Models 1.a, 1.b, and 1.c investigated the associations between BMI, WHR, and WC (respectively) and CBF_GM_ using fixed entry multivariable linear regression models controlling for age and sex. These relationships were further examined in models 2.a, 2.b, and 2.c which further adjusted for known risk factors. Existing literature was used to define an a priori set of additional potential correlates of CBF namely: PA, education, number of cardiovascular conditions (angina; high blood pressure; heart: attack ever, failure, murmur, abnormal rhythm; high cholesterol, TIA ever; grouped into a categorical variable 0, 1, or ≥2 conditions), diabetes, antihypertensive medication, alcohol, smoking, and depression.(Alosco et al., 2012; Birdsill et al., 2013; Williamson et al., 2018) In these multivariable analyses, BMI, WHR, and WC were each investigated independently in separate models due to collinearity. For all the above models standardized coefficients were also calculated as

(1) $$\frac{X-\bar{X}}{\text{SD}\left(X\right)},$$

where X is the measure of interest for a particular individual, $\bar{X}$ is the mean and SD(X) is the standard deviation across the cohort for the measure of interest. This was done in order to access effect sizes relative to the study cohort distribution, facilitating the comparison of results within and between models. Additionally, in order to further explore the influence of PA on these relationships, in models 3.a, 3.b, and 3.c the cohort was grouped by recommended BMI, WHR, and WC cut-offs and these groups further stratified by either low, moderate, or high PA levels. This was investigated using the same multivariable regression models described above (model 2.a−c), with groupings treated as categorical variables and the ‘normal’ ranges for BMI, WHR and WC taken as the control group in each respective model (the forming of these groups is illustrated in supplemental Figure A.1). Finally, to explore the potential effects of differing WHR within BMI groupings, BMI groups were further stratified by WHR cut-offs and the same fully controlled multivariable regression model was used; these results are presented as a supplemental to this study.

Statistical analysis was performed using *STATA 15.1* (StataCorp, USA). Normality of continuous variables was evaluated by visual assessment of curves. All multivariable analysis was completed using linear regression with residual analysis completed to assess model assumptions. All tests were 2-sided and *p* <.05 was considered statistically significant. Results from absolute coefficients are given as point estimates in appropriate units and results from standardized coefficient (*z*-score) analysis per SD as accessed for the cohort, all presented with 95% confidence intervals (CI).

### 2.6 Data availability statement

The datasets generated during and/or analyzed during the current study are not publicly available due to data protection regulations but are accessible at TILDA on reasonable request.

## 3 Results

### 3.1 Participant characteristics

Participants’ mean (SD) age at scan was 69.0 (7.4) years and 258 (52.1%) participants were female. Mean (SD) BMI, WHR, and WC were 28.0 (4.2) kg/m^2^, 0.91 (0.09), and 95.6 (12.9) cm respectively. By BMI, 378 (71.3%) participants were classified overweight/obese, while 340 (68.7%) and 381 (77.0%) were above the WHO cut-offs for WHR and WC respectively. Mean (SD) CBF_GM_ was 36.8 (8.1) ml/100g/min. High PA was reported by 131 (26.5%) participants, while 179 (36.1%) and 185 (37.4%) reported low or moderate PA respectively. Full demographic characteristics of the study cohort as well as the full TILDA Wave 3 cohort are presented in Table 2.

**Table 2:** Demographics and health characteristics of TILDA wave 3 and study sample

	TILDA W3 Cohort (N = 5,134^a^)	Study Cohort (N = 495)
Age [years]	66.2 (SD: 9.2, range: [37 − 96])^b^	69.0 (SD: 7.4, range: [50 − 92])
Sex [% (n)]	Female: 55.6% (2,854)	Female: 52.1% (258)
Education [% (n)]Primary/NoneSecondaryThird/Higher	23.6% (1,210)39.0% (2,003)37.4% (1,921)	21.2% (105)35.6% (176)43.2% (214)
Mean body mass index (BMI) [kg/m^2^]Underweight (BMI < 18.5)^c^ [% (n)]Normal (18.5 ≤ BMI < 25)^c^ [% (n)]Overweight (25 ≤ BMI < 30)^c^ [% (n)]Obese (BMI ≥ 35)^c^ [% (n)]	28.6 (SD: 5.2, range: [15.8 – 83.9])0.7% (38)22.7% (1,163)43.2% (2,216)33.5% (1,717)	28.0 (SD: 4.2, range: [18.8 – 45.8])-23.7% (117)46.9% (232)24.4% (146)
Mean Waist-to-Hip Ratio (WHR)<0.90 (M); <0.85 (W)^c^ [% (n)]≥0.90 (M); ≥0.85 (W)^c^ [% (n)]	0.91 (SD: 0.09, range: [0.48 – 1.23])31.1% (1,568)68.9% (3,536)	0.91 (SD: 0.09, range: [0.48 – 1.16])31.3% (155)68.7% (340)
Mean waist circumference (WC) [cm]<94 cm (M); <80 cm (W)^c^ [% (n)]≥94 cm (M); ≥80 cm (W)^c^ [% (n)]	96.2 (SD: 13.9, range: [59 – 163])21.1% (1,082)78.9% (4,052)	95.6 (SD: 12.9, range: [67 – 145])23.0% (114)77.0% (381)
Physical activity (IPAQ) [% (n)]LowModerateHigh	38.3% (1,965)35.1% (1,804)26.6% (1,365)	36.1% (179)37.4% (185)26.5% (131)
Self-reported diabetic [%]	8.5% (438)	7.9% (39)
Number of cardiovascular conditions^d^ [% (n)]012+	37.8% (1,941)35.3% (1,813)26.9% (1,380)	41.2% (204)34.6% (171)24.2% (120)
Antihypertensive medication Use^e^ [% (n)]	43.0% (2,206)	40.4% (200)
CAGE alcohol scaleCAGE < 2CAGE ≥ 2No response	74.7% (3,835)10.8% (555)14.5% (744)	79.8% (395)8.9% (44)11.3% (56)
Smoker [% (n)]NeverPastCurrent	45.5% (2,336)42.8% (2,196)11.7% (602)	52.3% (259)41.0% (203)6.7% (33)
CESD [% (n)]Non-depressed (CESD <9)Depressed (CESD ≥9)	86.5% (4,443)13.5% (691)	90.5% (448)9.5% (47)
MMSE [no. of correct responses]	28.6 (SD: 1.9, range: [6 – 30])	28.8 (SD: 1.5, range: [21 – 30])
Mean CBF [ml/100g/min]	-	36.5 (SD: 8.1, range: [13.9 – 66.4])

a Cohort wherein all measures available from home or health center assessment.

b TILDA also included spouses of participants, some of whom were under 55 years of age.

c World Health Organization cut-off points. (1995; Consultation, 2008)

d Cardiovascular conditions: angina; high blood pressure; heart: attack ever, failure, murmur, abnormal rhythm; high cholesterol, TIA ever.

e Coded using the Anatomical Therapeutic Chemical Classification (ATC): antihypertensive medications (ATC C02), diuretics (ATC C03), β-blockers (ATC C07), calcium channel blockers (ATC C08), and renin-angiotensin system agents (ATC C09) Abbreviations: IPAQ, international physical activity questionnaire; CESD, Center for Epidemiologic Studies Depression scale; MMSE, mini-mental state examination; CBF, cerebral blood flow.

### 3.2 Association of BMI, WHR, and WC with CBF

**Fig. 2:**
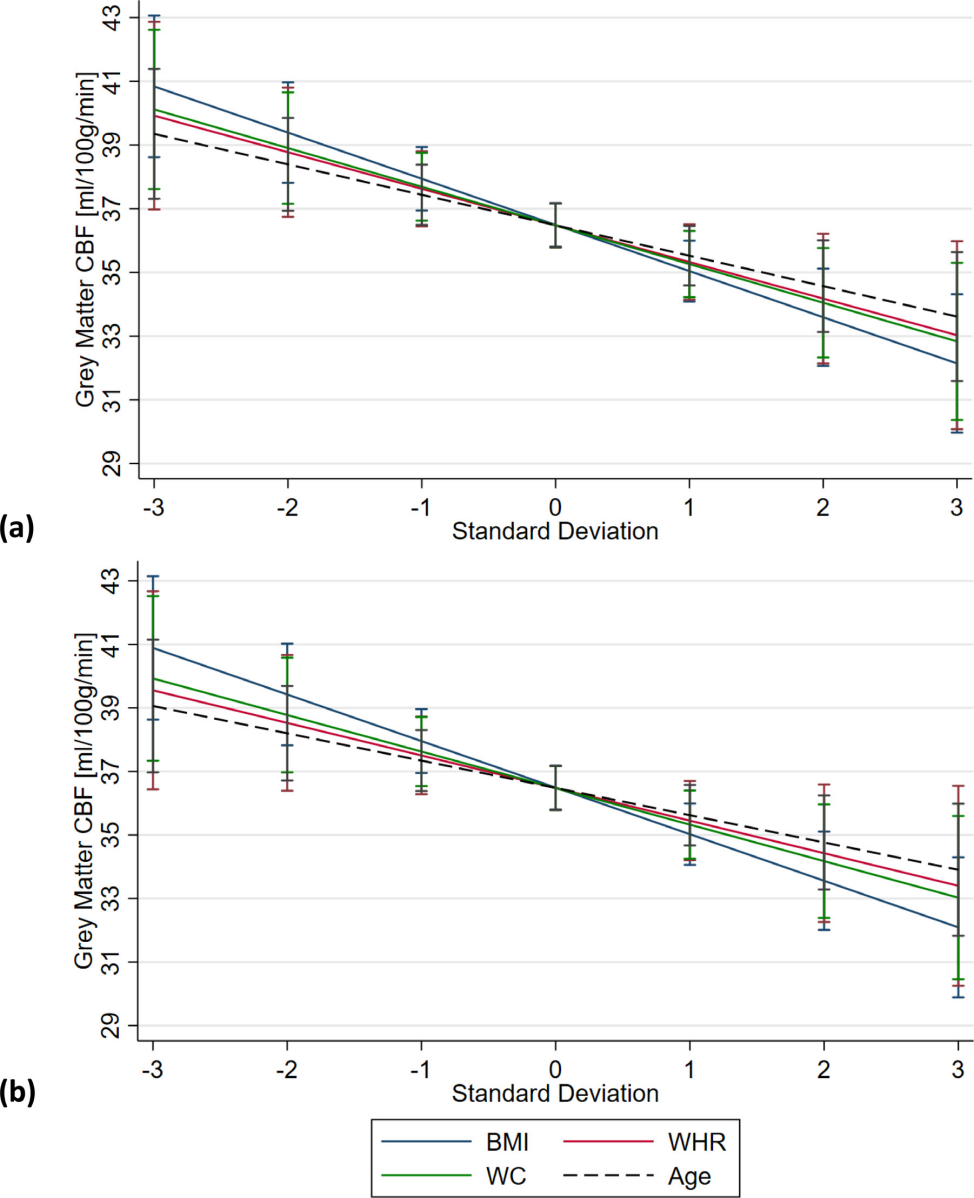
Predictive margin plots illustrating the regression results from (a) models 1.a-c and (b) models 2.a-c for the associations of obesity metrics (BMI, WHR and WC), as well as age, with gray matter cerebral blood flow Data are presented across ± 3 standard deviations of the cohort distribution. (A) Models 1.a, 1.b, and 1.c: association of mean body mass index (BMI), waist-to-hip ratio (WHR), and waist circumference (WC) with gray matter cerebral blood flow (CBFGM [ml/100g/min]) adjusted for age and sex. (B) Models 2.a, 2.b, and 2.c: association of BMI, WHR, and WC with CBFGM fully adjusted for all corelates. Age associations taken from models (A) 1.b and (B) 2.b. Error bars represent the 95% CIs, shown at the mean and at ± 1, 2 and 3 standard deviations from the mean for each measure (BMI, WHR, WC and age).

In models 1.a, 1.b, and 1.c higher BMI (β = -0.34 ml/100g/min per 1 kg/m^2^, [95% CI, -0.51 to -0.18], *P* < 0.001), WHR (β = -1.29 ml/100g/min per 0.1 WHR, [95% CI, -2.38 to -0.22], *p* = 0.019), and WC (β = -0.13 ml/100g/min per 1 cm, [95% CI, -0.22 to -0.05], *p* =0 .003) were all associated with reduced CBF_GM_. In all three models, CBF_GM_ was reduced with increasing age by 0.13 − 0.15 ml/100g/min per 1 year ([combined 95% CI, -0.24 − -0.04], *p* ≤ 0.003). Females had higher CBF_GM_ in all three models (model 1.a (BMI): β = 2.84 ml/100g/min [95% CI, 1.47 − 4.21], *p* < 0.001; model 1.b (WHR): β = 1.78 ml/100g/min [95% CI, 0.07 − 3.49], *p* = 0.041; model 1.c (WC): β = 2.05 ml/100g/min [95% CI, 0.54 − 3.56], *p* = 0.008). Comparing the standardized coefficients (z-scores), BMI was associated with a greater reduction in CBF_GM_ (β = -1.45 ml/100g/min per 1 SD [95% CI, -2.15 − -0.75], *p* ≤ 0.001) than that of WHR (β = -1.15 ml/100g/min per 1 SD [95% CI, -2.10 − -0.19], *p* = 0.019) and WC (β = -1.21 ml/100g/min per 1 SD [95% CI, -2.01 − -0.42], *p* = 0.003). All three obesity metrics were associated with a larger reduction in CBF_GM_ compared with that of age (β = -0.93 to -1.08 ml/100g/min per 1 SD [combined 95% CI, -1.73 to -0.27], *p* ≤ 0.006), as illustrated in Fig. 2(a). Results from models 1.a, 1.b, and 1.c are presented in Table 3.

**Table 3:** Association of mean body mass index (BMI), waist-to-hip ratio (WHR), and waist circumference (WC) with gray matter cerebral blood flow (CBF_GM_)

	β (95% CI)	*p* Value
CBF_GM_ (N = 495) [ml/100g/min]		
Model 1.a		
BMI [per 1 kg/m^2^][Z-score: per 1 SD]	-0.34 (-0.51 to -0.18)-1.45 (-2.15 − -0.75)	<0.001
Age [per 1 year][Z-score: per 1 SD]	-0.15 (-0.24 − -0.07)-1.11 (-1.74 − -0.49)	0.001
Sex [Female]	2.84 (1.47 to 4.21)	<0.001
Model 1.b		
WHR [per 0.1][Z-score: per 1 SD]	-1.29 (-2.38 − -0.22)-1.15 (-2.10 − -0.19)	0.019
Age [per 1 year][Z-score: per 1 SD]	-0.13 (-0.22 − -0.04)-0.96 (-1.59 − -0.32)	0.003
Sex [Female]	1.78 (0.07 − 3.49)	0.041
Model 1.c		
WC [per 1 cm][Z-score: per 1 SD]	-0.13 (-0.22 − -0.05)-1.21 (-2.01 − -0.42)	0.003
Age [per 1 year][Z-score: per 1 SD]	-0.13 (-0.22 − -0.05)-0.96 (-1.59 − -0.33)	0.003
Sex [Female]	2.05 (0.54 − 3.56)	0.008
Model 2.a		
BMI [per 1 kg/m^2^][Z-score: per 1 SD]	-0.35 (-0.51−-0.18)-1.47 (-2.17 − -0.76)	<.001
Age [per 1 year][Z-score: per 1 SD]	-0.15 (-0.23 − -0.06)-1.07 (-1.72 − -0.42)	0.001
Sex [Female]	2.64 (1.18 − 4.11)	< .001
Model 2.b		
WHR [per 0.1][Z-score: per 1 SD]	-1.15 (-2.30 − -0.01)-1.03 (-2.04 − -0.01)	0.048
Age [per 1 year][Z-score: per 1 SD]	-0.12 (-0.21 − -0.03)-0.86 (-1.51 − -0.20)	0.010
Sex [Female]	1.77 (-0.05 − 3.59)	0.056
Model 2.c		
WC [per 1 cm][Z-score: per 1 SD]	-0.09 (-0.15 − -0.03)-1.15 (-1.98 − -0.32)	0.007
Age [per 1 year][Z-score: per 1 SD]	-0.12 (-0.21 − -0.03)-0.88 (-1.54 − -0.23)	0.008
Sex [Female]	1.94 (0.32 − 3.55)	0.019

Models 1.a, 1.b, and 1.c are adjusted for age and sex. Models 2.a, 2.b, and 2.c are adjusted for age, sex, physical activity (categorized by the international physical activity questionnaire (IPAQ)), education, diabetes, number of cardiovascular conditions (angina; high blood pressure; heart: attack ever, failure, murmur, abnormal rhythm; high cholesterol, TIA ever. Grouped into a categorical variable 0, 1, or ≥2 conditions), antihypertensive medication use (coded using the Anatomical Therapeutic Chemical Classification (ATC): antihypertensive medications (ATC C02), diuretics (ATC C03), β-blockers (ATC C07), calcium channel blockers (ATC C08), and renin-angiotensin system agents (ATC C09)), alcohol consumption, smoking, and depression. Standardized coefficients (*z*-scores) also presented for continuous variables. Abbreviations: CI, confidence intervals; BMI, body mass index; WHR, waist-to-hip ratio; WC, waist circumference.

In models 2.a, 2.b, and 2.c, which controlled for additional risk factors, all obesity indicators retained significant associations with reduced CBF_GM_ (BMI: β = -0.35 ml/100g/min per 1 kg/m^2^, [95% CI, -0.51 to -0.18], *p* < 0.001; WHR: β = -1.15 ml/100g/min per 0.1, [95% CI, -2.30 − -0.01], *p* = 0.048; and WC: β = -0.09 ml/100g/min per 1 cm, [95% CI, -0.15 − -0.03], *p* = 0.007). In all models increased age was associated with a reduction in CBF_GM_ of between 0.12 and 0.15 ml/100g/min per 1 year ([combined 95% CI, -0.23 − -0.03], *p* ≤ 0.010). Comparing obesity metrics via standardized coefficients (z-scores), as with models 1.a.–1.c BMI was found to correlate with a greater reduction in CBF_GM_ than WHR or WC, with an increase of 1 SD in BMI being associated with a reduction in CBF_GM_ of 1.47 ml/100g/min ([95% CI, -2.17 − -0.76], *p* < 0.001), compared with 1.01 ml/100g/min ([95% CI, -2.04 − -0.01], *p* = 0.048) and 1.15 ml/100g/min ([95% CI, -1.98 − -0.32], *p* = 0.007) for WHR and WC respectively. BMI, WHR, and WC were again associated with a larger reduction in CBF_GM_ (β = -1.01 − -1.47 ml/100g/min per 1 SD [combined 95% CI, -2.17 − -0.01], *p* ≤ 0.048) compared with that of age (β = -0.86 to -1.07ml/100g/min per 1 SD [combined 95% CI, -1.72 to -0.20], *P* ≤ .010), as shown in Fig. 2(b). Females had higher CBF_GM_ in model 2.a (BMI: β = 2.64 ml/100g/min [95% CI, 1.18 to 4.11], *P* < .001) and model 2.c (WC: β = 1.94 ml/100g/min [95% CI, 0.32 to 3.55], *P* = .019). No significant associations were found for the other risk factors investigated in these multivariable analyses. Results from models 2.a, 2.b, and 2.c are presented in Table 3.

**Fig. 3:**
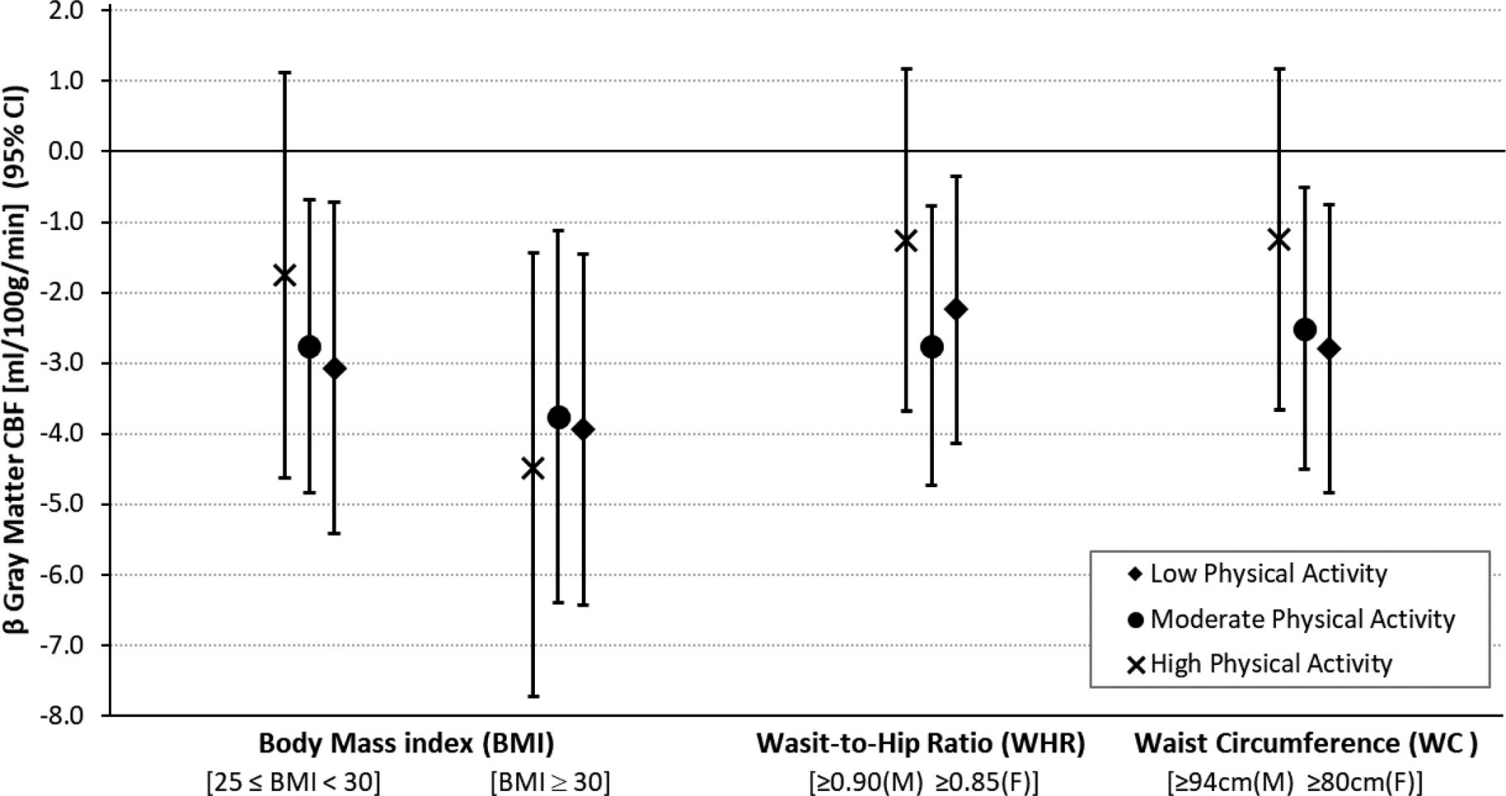
Plots illustrating the group analysis results from models 3.a, 3.b, and 3.c. Plots show association of body mass index (BMI), waist-to-hip ratio (WHR), and waist circumference (WC) cut-off groups (stratified by the international physical activity questionnaire (IPAQ) groups) with gray matter cerebral blood flow [ml/100g/min] fully adjusted for all corelates. Results presented are β coefficients from regression models c.1-3 and error bars represent the 95% confidence interval from these regression models. All results presented are verses ‘normal’ BMI, WHR, or WC groups. If error bars cross the “0” line results are not significant to the set significance level of *p* < 0.05. For interpretation of the references to color in this figure legend, the reader is referred to the Web version of this article.

In model 3.a no significant association was found for those reporting high PA within this BMI-defined overweight group, however, a combination of being overweight by BMI and reporting low/moderate PA was associated with lower CBF_GM_ compared with the control group (Moderate: β = -2.76 ml/100g/min [95% CI, -4.83 to -0.69], *P* = .009; Low: β = -3.07 ml/100g/min [95% CI, -5.41 to -0.72], *P* = .011). For those classified as obese by BMI all three PA groups were associated with reduced CBF_GM_ (High: β = -4.48 ml/100g/min [95% CI, -7.54 − -1.44], *p* = 0.004; Moderate: β = -3.76 ml/100g/min [95% CI, -6.40 − -1.12], *P* = .005; Low: β = -3.94 ml/100g/min [95% CI, -6.43 − -1.46], *p* = 0.002). In Model 3.b groups with high WHR and low or moderate levels of PA were associated with reduced CBF_GM_ (Moderate: β = -2.76 ml/100g/min [95% CI, -4.74 to -0.78], *p* = 0.006; Low: β = -2.24 ml/100g/min [95% CI, -4.13 − -0.35], *p* = 0.020). However, a combination of high WHR and high PA was not significantly associated with a reduction in CBF_GM_. Similarly, for WC groups (Model 3.c) combined high WC and low or moderate PA were associated with the reduced CBF_GM_ (Moderate: β = -2.51 ml/100g/min [95% CI, -4.51 − -0.51], *p* = 0.014; Low: β = -2.79 ml/100g/min [95% CI, -4.83 − -0.75], *p* = 0.007). A combination of high WC and high PA was not significantly associated with reduced CBF_GM_. The results of this group analysis are presented in Table 4 and illustrated in Fig. 3.

**Table 4:** Association of body mass index (BMI), waist-to-hip ratio (WHR), and waist circumference (WC) cut-off groups (stratified by physical activity) with gray matter cerebral blood flow (CBF_GM_)

		β (95% CI)	*p* Value
CBF_GM_ (N = 495) [ml/100g/min]			
Model 3.a			
BMINormal (Reference)Overweight[25 ≤ BMI < 30]^a^Obese/ M. Obese[BMI ≥ 30]^a^	Physical Activity (IPAQ)(N = 117)High (N = 59)Moderate (N = 96)Low (N = 77)High (N = 36)Moderate (N = 48)Low (N = 62)	-1.75 (-4.63 − 1.13)-2.76 (-4.83 − -0.69)-3.07 (-5.41 − -0.72)-4.48 (-7.53 − -1.44)-3.76 (-6.40 − -1.12)-3.94 (-6.43 − -1.46)	0.2340.0090.0110.0040.0050.002
Age [per 1 year]		-0.14 (-0.23 − -0.06)	0.001
Sex [Female]		2.49 (1.02 − 3.95)	0.001
Model 3.b			
WHRNormal (Reference)Overweight/Obese[≥0.90 (M)≥0.85 (F)]^a^	Physical Activity (IPAQ)(N = 155)High (N = 91)Moderate (N = 119)Low (N = 130)	-1.26 (-3.69 − 1.17)-2.76 (-4.74 − -0.78)-2.24 (-4.13 − -0.35)	0.3070.0060.020
Age [per 1 year]		-0.11 (-0.20 − -0.03)	0.011
Sex [Female]		2.25 (0.75 − 3.76)	0.003
Model 3.c			
WCNormal (Reference)Overweight/Obese[≥0.94 cm (M)≥0.80 cm (F)]^a^	Physical Activity (IPAQ)(N = 114)High (N = 93)Moderate (N = 145)Low (N = 143)	-1.25 (-3.66 − 1.18)-2.51 (-4.51 − -0.51)-2.79 (-4.83 − -0.75)	0.3130.0140.007
Age [per 1 year]		-0.12 (-0.21 − -0.03)	0.008
Sex [Female]		2.95 (1.48 − 4.43)	<0 .001

Key: BMI, body mass index; CI, confidence intervals; IPAQ, International physical activity questionnaire; WC, waist circumference; WHR, waist-to-hip ratio.

a World Health Organization recommended cut-off points. (1995; Consultation, 2008) Models 3.a, 3.b, and 3.c are adjusted for age, sex, education, diabetes, number of cardiovascular conditions (angina; high blood pressure; heart: attack ever, failure, murmur, abnormal rhythm; high cholesterol, TIA ever. Grouped into a categorical variable 0, 1, or ≥2 conditions), antihypertensive medication use (coded using the Anatomical Therapeutic Chemical Classification (ATC): antihypertensive medications (ATC C02), diuretics (ATC C03), β-blockers (ATC C07), calcium channel blockers (ATC C08), and renin-angiotensin system agents (ATC C09)), alcohol consumption, smoking, and depression.

Results from the same fully controlled multivariable regression model used above, but with BMI groups further stratified by WHR cut-offs are presented in Appendix A, Table A1. This analysis revealed that having BMI within recommended limits (‘normal’ group) but WHR above recommended cut-offs was not associated with reduced CBF_GM_, relative to the reference group (i.e. both BMI and WHR within recommended levels). For those overweight by BMI, also having WHR above cut-offs was associated with greater reduction in CBF_GM_ than those with low WHR, relative to the reference group (BMI: overweight - WHR below cut-offs: β = -3.01 ml/100g/min [95% CI, -5.78 to -0.24], *p* = 0.033 and WHR above cut-offs: β = -3.41 ml/100g/min [95% CI, -5.74 − -1.09], *p* = 0.004). For participant obese by BMI, but with WHR below recommended cut-offs, no significant reduction in CBF_GM_ was observed; those both obese by BMI and having high WHR had the largest reduction in CBF_GM_ for this model (β = -4.83 ml/100g/min [95% CI, –7.25 − -2.41], *p* < 0.001).

## 4 Discussion

In this study we observed that overweight and obesity were associated with reduced CBF_GM_ with all obesity measures (BMI, WHR and WC) associated with a similar change in CBF_GM_. The magnitude of obesity associations with CBF_GM_ was demonstrated to be stronger than that of age across the range of the cohort. An increase in WC of 1.3 cm associated with the same reduction in CBF_GM_ as 1 year of advancing age. This study also demonstrated that higher levels of physical activity may ameliorate the association of overweight and obesity with reduced CBF_GM_.

Previous studies have suggested that obesity in early and midlife may be linked with reduced cerebral perfusion and blood flow velocity,(Willeumier et al., 2011; Williamson et al., 2018) however, little is known about these associations in an aged population.(Birdsill et al., 2013) To date, studies investigating cerebral blood flow, particularly in in older adults, have been limited by small cohort sizes, as well as limitations with the imaging modalities used; however, previously reported results are in line with the present work with regards obesity and cerebral haemodynamic associations.(Alosco et al., 2012; Birdsill et al., 2013; Képes et al., 2021; Selim et al., 2008; Willeumier et al., 2011)

Birsill et al. in a 2013 study of 69 individuals (mean age = 60.4 ± 6.1 years) examined associations between metabolic syndrome (which included a measure of central obesity; WC) and CBF_GM_. The study reporting that central obesity (WC) might potentially be a strong metabolic risk factor for reducing CBF_GM_.(Birdsill et al., 2013) However, as the authors note themselves, the small sample was limiting, and replication in a larger sample would be required to verify the results. This study, as with the present work, utilized pCASL-MRI; a non-invasive, quantitative method capable of calculating whole-brain perfusion. This imaging technique employs magnetically-labeled arterial blood as an endogenous tracer therefore requiring no exogenous contrast agent to be administered and imparts no ionizing radiation to the subject; potentially providing an optimal method for population-based, as well as clinical CBF assessment.(Fan et al., 2016; Wintermark et al., 2005) High concordance rates have been reported between pCASL-MRI and [15O]-water positron emission tomography (PET), which is considered the reference standard for cerebral perfusion measurement, however PET is invasive, requires the use of ionizing radiation, and is more expensive than MRI.(Fan et al., 2016)

Alosco et al. reported a significant interaction between cerebral blood flow velocity and BMI (N = 99; mean age = 67 ± 11 years), suggesting that a combination of hypoperfusion and high BMI had an adverse influence on attention and executive function in heart failure patients.(Alosco et al., 2012) In a 2008 study of 137 individuals (age = 50 − 85 years), Selim et al. reported that higher BMI was associated with lower mean blood flow velocities, independent of diagnosis of diabetes, hypertension, or stroke.(Selim et al., 2008) Both of these studies employed transcranial Doppler ultrasound, a commonly used imaging modality for the assessment of cerebral hemodynamic. However, this technique is highly operator dependent and measurements are limited to the large basal arteries, providing only an index of global rather than local cerebral blood flow velocity, it also does not provide a measure of cerebral perfusion.(Purkayastha and Sorond, 2012)

Willeumier et al. in a 2011 study of 36 individuals (mean age = 60.4 ± 6.1 years) utilized a combination of single photon emission computed tomography (SPECT) and statistical parametric mapping (SPM). The study reported that higher BMI in healthy individuals was associated with decreased regional CBF in Broadmann areas 8, 9, 10, 11, 32, and 44, brain regions involved in attention, reasoning, and executive function.(Willeumier et al., 2011) In a recent study by Képes et al. of 26 individuals (median age = 53.5 ± 9.9 years) BMI was also reported to be significantly associated with brain perfusion measured using SPECT.(Képes et al., 2021) There are however several limitations to SPECT imaging, including that protocols tend to be lengthy and inefficient, potentially unreliable supply of Tc-99m, it requires the use of ionizing radiation and can be prohibitively expensive.(Bateman, 2012)

Consistent with prior studies using various imaging modalities,(Chen et al., 2011; Leidhin et al., 2021; Liu et al., 2012; Lu et al., 2011; Parkes et al., 2004; Selim et al., 2008; Wolters et al., 2017; Zhang et al., 2018) in the present study an age-related decrease in CBF_GM_ was found for all models. Direct comparison between the present work and previous studies is limited due to significant differences in methodologies. However, the present study followed best-practice consensus guidelines for 3T pCASL-MRI (Alsop et al., 2015) and absolute CBF results are in line with previous studies where CBF was measured using 3T pCASL-MRI according to the same recommendations. Following a similar protocol to the one used herein, Jefferson et al. measured whole brain CBF values in 270 older adults (mean age = 73 ± 7 years), reporting mean values of 37.3 ± 7.1 ml/100g/min.(Jefferson et al., 2018) In another 3T pCASL MRI study, Chen et al. reported that mean cortical CBF_GM_ decreased with age, reporting values of 52 ± 10.7 and 42.7 ± 8.8 ml/100g/min in middle-aged (mean age 52 ± 6 years, n = 38) and older adults (mean age 71 ± 10 years, n = 37) respectively.(Chen et al., 2011) It has previously been suggested that age-related decrease in cerebral perfusion could potentially be a consequence of cerebral atrophy, and/or decreased neuronal/metabolic activity and demand.(Parkes et al., 2004; Zhang et al., 2017) However, this hypothesis is controversial, as other studies have reported regional reductions in CBF to be independent of age-related atrophy.(Chen et al., 2011) The Rotterdam study demonstrated the potentially bidirectional nature of this relationship, reporting that smaller baseline brain volume resulted in steeper decrease in CBF over time. However, the same study also reporting that lower CBF at baseline was associated with accelerated brain atrophy, though only in subjects aged 65 years or older.(Zonneveld et al., 2015) This finding suggests that the relationship between CBF and atrophy becomes more complex as a population ages. In the present study it was demonstrated that anthropometric obesity measures were associated with a larger reduction in CBF_GM_ than age in an older cohort. In absolute terms an increase in BMI of 0.43 kg/m^2^, WHR of 0.01, or WC of 1.3 cm correlated with the same reduction in CBF_GM_ associated with 1 year of advancing age.

Sex differences were observed in most models (with the exception of the multivariable continuous WHR model (model 2.b)); this is not unexpected and is in line with previous studies where it was likewise found that global CBF was increased in women.(Leidhin et al., 2021; Liu et al., 2012; Zhang et al., 2018) Indeed, in a recent study reporting normative CBF_GM_ values stratified by sex, not only were women found to have higher CBF_GM_ overall on average, as reported herein, but also a slower decline in CBF_GM_ between the ages of 54 and 84 years.(Leidhin et al., 2021) Additionally, normative brain volume values from the Framingham Heart study reported sex differences in brain volumes, with women having modestly larger total brain and frontal lobe volumes (normalized to head size) across all age groups investigated, as well as larger volumes of white matter hyperintensities. Also, similar to CBF, males were reported as having a faster decline in brain volume over time.(DeCarli et al., 2005) With the results from the current study, although we observed relatively large sex differences, these differences did not appear to have influenced the associations of obesity / PA with CBF_GM_ (a separate sub-analysis was ran which interacted sex with the obesity / PA groups used in models 3.a-c and no significant interactions were observed; results not presented herein). One potential explanation for these observed sex differences could be that generally women have lower haematocrit than men resulting in reduced oxygen carrying capacity necessitating higher CBF to supply the brain with the required oxygen.(Zeng et al., 2001) Another plausible reason could be differences in sex hormones between women and men, for example estradiol and estrogen levels which influence, among other things, vascular endothelial growth factor (VEGF), as well as potentially affecting brain development over the lifecourse.(DeCarli et al., 2005; Mueller et al., 2000) These differences in CBF may also be related to underlying sex-related differences in neurological disease development with age.(DeCarli et al., 2005)

The specific mechanisms underlying the association between obesity and reduced CBF_GM_ are not well understood. One possible hypothesis is that the increased secretion of pro-inflammatory cytokines such as tumor necrosis factor alpha (TNF-α) or Interleukin 6 (IL-6) from fat deposits contributes to inflammation and localized tissue damage in the brain and surrounding tissue/blood vessels. This theory could be supported by the results presented in Appendix A, where it was found that the combination of overall obesity (BMI ≥30 kg/m^2^) and central obesity (WHR >0.85 for women and >0.90 for men) was associated with reduced CBF_GM_, but not overall obesity without central obesity, compared with CBF_GM_ measured in lean participants; centrally-located visceral fat is thought to be a major site for inflammatory cytokine production and has been previously been linked to other vascular risk factors and brain atrophy.(Després, 2006; Hamer and Batty, 2019) However, the small group sizes available for this analysis mean that these results should be treated with caution and will require further, larger scale studies to explore this potential mechanistic path further. Another plausible supposition is that the negative physiological and mechanical effects of obesity on cardiovascular function may contribute to impaired CBF. Obesity has been demonstrated to be associated with a lower brain volume,(Climie et al., 2015; Hamer and Batty, 2019) and decline in brain volume is associated with reduced CBF.(Zonneveld et al., 2015) However, the causal direction for this association (brain atrophy and CBF) in older adults remains elusive, as discussed further below, and will require future longitudinal studies to elucidate.(Zonneveld et al., 2015)

In the current study, for individuals overweight by BMI or above WHR/WC cut-offs, high levels of PA reduced/removed the associations with reduced CBF_GM_. Previous research has shown longitudinally that early-life PA can influence later-life neurocardiovascular health. For example, in a study of young army recruits (N = 1.1 million), aerobic fitness at age 18 was predictive of future dementia risk in older age.(Nyberg et al., 2014) Another study of middle-aged Swedish females found that those who had a high level of fitness at baseline had an eight times lower risk of dementia at 44-year follow-up.(Hörder et al., 2018) Hence, it is unclear whether the observed relationships in the current study are due to current lifestyle habits, or long-term higher levels of PA; further work is required to expound this relationship. Potential mechanisms to explain these results may include the ability of PA to slow down the accumulation of visceral fat and subsequent chronic systemic inflammation.(Pedersen, 2019) PA also significantly improves all-body cardiovascular fitness with increased blood flow and improved mitochondrial function.(Irving et al., 2015) These positive benefits could facilitate neuroprotection and neuroplasticity in the brain through increased production of neurotropic factors.(Ahlskog et al., 2011; Christie et al., 2008)

This study has several limitations which should be kept in mind when interpreting the results. Firstly, since this study was cross-sectional, causality or even temporality of the observed relationships could not be inferred. Second, PA was self-reported and thereby not an objective measure of activity, however, IPAQ is a widely used and accepted method for quantifying PA in epidemiological studies. Limitations with regards IPAQ’s specificity may be the reason that there were unclear trends with regards dose/response for some of the models used here (e.g. models 3.a and 3.b). Future work using more objective PA measures would hopefully shed further light on this relationship. Third, there were only two underweight individuals in the MRI cohort who were excluded from this analysis; since it has been previously demonstrated that being underweight is also associated with higher risk of vascular dementia,(Lee et al., 2020) a future similar study investigating underweight individuals would be of interest. Although persons with self-reported history of neurological conditions (stroke and Parkinson’s disease) were excluded from this study, no specific exclusions were applied for poor cognitive performance, however, the cohort had good performance overall on the MMSE, with only 5 participants scoring < 24, and none scoring < 21. In this study CBF_GM_ was assessed as a marker of global cerebral perfusion, as this is known to provide a good indication of overall cerebrovascular heath. However, it is worth noting that global CBF may lack specificity in the readout. For example, in previous works examining regional age-related differences in CBF, with increasing age, regions of both increased and decreased perfusion were reported. For instance, as age increases blood flow to the parietal cortex and precuneus may be reduced, while other regions, such as the temporal lobe, posterior and anterior cingulate cortex may increase.(Lee et al., 2009; Preibisch et al., 2011; Zhang et al., 2018) Finally, longitudinal follow-up will be required to determine the clinical significance of the observed findings and as such, this study should be considered preliminary and exploratory but does support a need for future work.

## 5 Conclusion

In this study involving older adults, higher BMI, WHR, and WC were associated with reduced CBF_GM_. The magnitude of this association was found to be greater than that of age. Increased PA was also shown to potentially protect against reduced CBF_GM_ in certain overweight/centrally-obese groups. CBF_GM_ was found to decrease with age and was significantly higher in women; these sex differences could potentially underpin or contribute to sex differences we observe in neurological disorder prevalence. Since cerebral hypoperfusion is an early mechanism in Alzheimer’s disease and vascular dementia, the findings of this study could inform the development of dementia prevention strategies. Further research is needed to validate these findings and determine their clinical implications.

## Disclosure statement

The authors have no actual or potential conflicts of interest.

## Credit Authorship

**Silvin P. Knight:** Conceptualization, Data curation, Formal analysis, Investigation, Methodology, Software, Visualization, Project administration, Roles/Writing - original draft, Writing - review & editing. **Eamon Laird:** Conceptualization, Methodology, Visualization, Roles/Writing - original draft, Writing - review & editing. **Wilby Williamson:** Formal analysis, Methodology, Writing - review & editing. **John O’Connor:** Formal analysis, Methodology, Software, Writing - review & editing. **Louise Newman:** Formal analysis, Methodology, Software, Writing - review & editing. **Daniel Carey:** Formal analysis, Methodology, Writing - review & editing. **Celine De Looze:** Project administration, Methodology, Writing - review & editing. **Andrew J. Fagan:** Project administration, Methodology, Writing - review & editing.

**Michael A. Chappell:** Methodology, Software, Writing - review & editing, Validation. **James F. Meaney:** Funding acquisition, Project administration, Resources, Supervision, Writing - review & editing. **Rose Anne Kenny:** Conceptualization, Funding acquisition, Project administration, Resources, Supervision, Writing - review & editing.

## Figures and Tables

**Table:** 

		β (95% CI)	*p* Value
CBF_GM_ (N = 495) [ml/100g/min]			
BMI^a^Normal (Ref.)Overweight[25 ≤ BMI < 30]^a^Obese/ M. Obese[BMI ≥ 30]^a^	WHR^a, b^Low (Ref. N = 76)High (N = 41)Low (N = 61)High (N = 171)Low (N = 18)High (N = 128)	-2.10 (-5.02 − 0.83)-3.01 (-5.78 − -0.24)-3.41 (-5.74 − -1.09)-3.94 (-8.63 − 0.76)-4.83 (-7.25 − -2.41)	0.1590.0330.0040.100< 0.001
Age [per 1 year]		-0.13 (-0.22 to -0.04)	.004
Sex [Female]		2.33 (0.81 to 3.85)	.003

Key: CI, confidence intervals.

^a^World Health Organization recommended cut-off points. (1995; Consultation, 2008) ^b^WHR cut-offs: ≥0.85 for females and ≥0.9 for males. Model adjusted for age, sex, education, diabetes, number of cardiovascular conditions (angina; high blood pressure; heart: attack ever, failure, murmur, abnormal rhythm; high cholesterol, TIA ever. Grouped into a categorical variable 0, 1, or ≥2 conditions), antihypertensive medication use (coded using the Anatomical Therapeutic Chemical Classification (ATC): antihypertensive medications (ATC C02), diuretics (ATC C03), β-blockers (ATC C07), calcium channel blockers (ATC C08), and renin-angiotensin system agents (ATC C09)), alcohol consumption, smoking, depression, and physical activity (IPAQ, International physical activity questionnaire).

## Appendix A

Table A1: Association between body mass index (BMI) groups, stratified by waist-to-hip ratio (WHR) cut-offs, and gray matter cerebral blood flow (CBF_GM_)

## Appendix B Supplementary materials

Image, application 1
