# Supplementary material for: Obesity is associated with reduced cerebral blood flow – modified by physical activity
Source: Neurobiol Aging. 2021 Sep;105:35–47. doi: 10.1016/j.neurobiolaging.2021.04.008 (PMC8600128; doi:10.1016/j.neurobiolaging.2021.04.008)

Figure A.1. Flow chart illustrating how the groups were formed for use as categorical variables in models 3.a-c

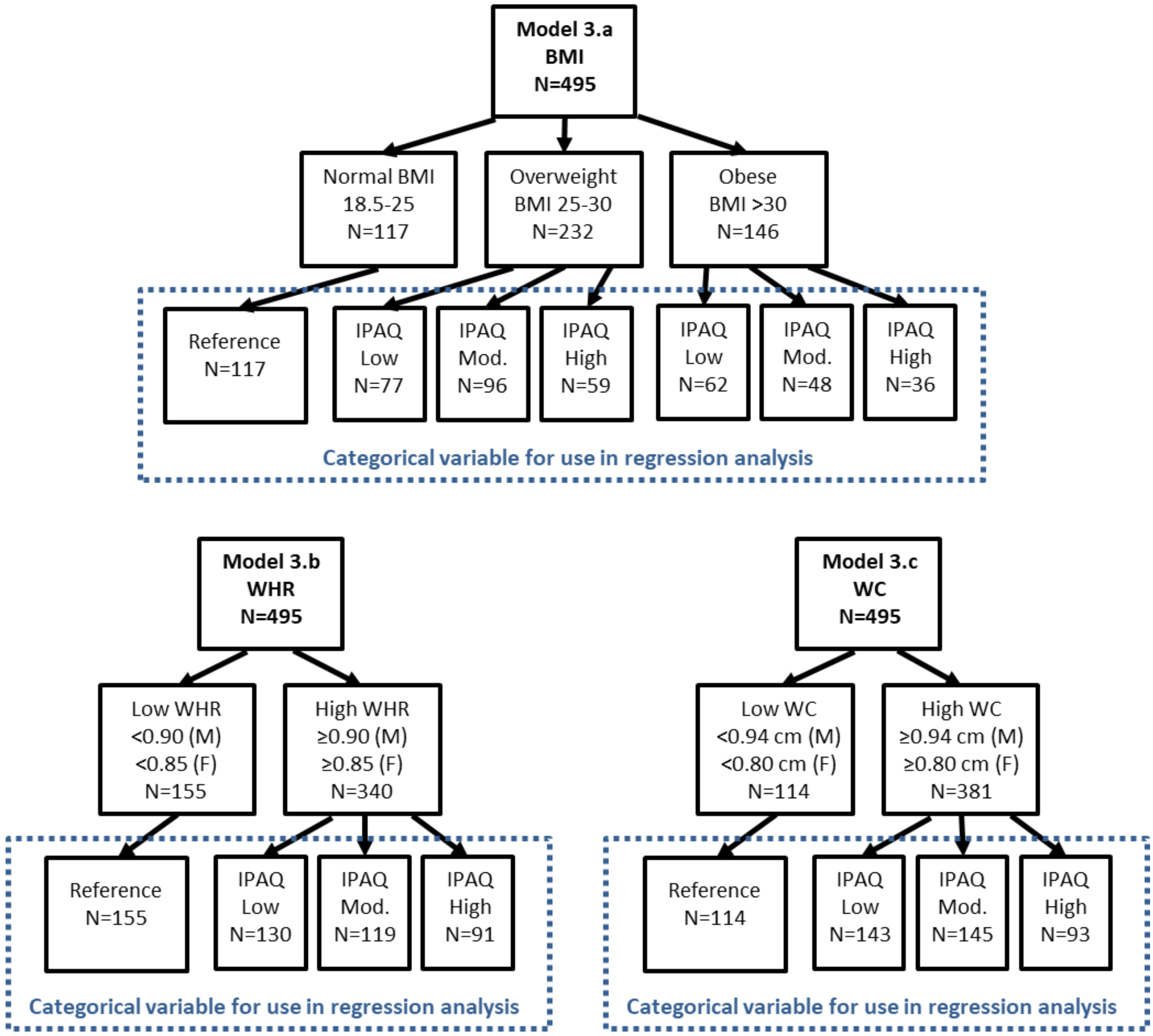

Supplement: Supplementary file 1 [file mmc1.pdf]
